# Differentiation of *Candida auris* from other pathogenic yeasts using near-infrared spectroscopy and multivariate analysis: a proof-of-concept study

**DOI:** 10.3389/fmicb.2026.1857107

**Published:** 2026-07-08

**Authors:** Maria R. C. Inácio, Ayrton L. F. Nascimento, Anthony G. J. Medeiros, Kássio M. G. Lima, Rafael W. Bastos

**Affiliations:** 1 Centro de Biociências, Universidade Federal do Rio Grande do Norte, Natal, Brazil; 2 Laboratório de Química Biológica e Quimiometria, Instituto de Química, Universidade Federal do Rio Grande do Norte, Natal, Brazil; 3 National Institute of Science and Technology in Human Pathogenic Fungi, Ribeirão Preto, Brazil

**Keywords:** chemometrics, diagnostic methods, fungal identification, infrared spectroscopy, mycology

## Abstract

**Introduction:**

*Candida* (*Candidozyma*) *auris* has emerged as a major public health concern due to its multidrug resistance, high mortality rates, and outbreak potential. These challenges are intensified by the difficulty of accurately identifying this species, particularly in settings with limited laboratory resources. This difficulty arises because *C. auris* is closely related to other yeast species, such as those within the *Candida haemulonii* complex. Although we previously demonstrated that near-infrared spectroscopy (NIRS) combined with multivariate analysis can discriminate *C. auris* from *C. haemulonii stricto* sensu, its performance against other clinically important yeasts had not been evaluated.

**Materials and methods:**

In this study, we assessed NIRS coupled with different multivariate analytical techniques as a tool for distinguishing *C. auris* from *C. haemulonii*, *C. albicans*, *C. tropicalis*, *C. parapsilosis*, *Nakaseomyces glabrata* (formerly *C. glabrata*), and *Pichia kudriavzevii* (formerly *C. krusei*). Each of the seven species was cultured on fifteen Sabouraud Dextrose agar plates at 37 °C. After 72 h, three isolated colonies per plate (45 colonies per species) were subjected to Fourier-transform NIR analysis, resulting in a total of 315 spectra. The spectra were preprocessed and analyzed using principal component analysis (PCA), successive projections algorithm (SPA), genetic algorithm (GA), and linear discriminant analysis (LDA) to construct classification models.

**Results and discussion:**

The combination of PCA, SPA, and GA with LDA achieved 100% sensitivity, specificity, and accuracy. These findings demonstrate that NIRS coupled with multivariate analysis can reliably differentiate *C. auris* from other medically important yeasts. The models also showed strong discriminatory capacity among the most prevalent pathogenic yeast species, reinforcing the promise of this approach as a rapid diagnostic tool for overcoming current identification challenges.

## Introduction

Species of the genus *Candida* (and related genera formerly classified within *Candida*) comprise a heterogeneous group of yeast-like fungi that may or may not undergo dimorphic transitions into pseudohyphal or hyphal forms. Although these fungi are part of the human microbiota, under certain conditions they can shift to a parasitic state, leading to infections collectively known as candidiasis (Csutak and Corbu, 2025; Mukaremera et al., 2017).

The most clinically relevant pathogenic species include *Candida albicans*, *Candida tropicalis*, *Candida parapsilosis*, *Nakaseomyces glabrata* (formerly *Candida glabrata*), and *Pichia kudriavzevii* (formerly *Candida krusei*) (Bastos et al., 2021). Historically, *C. albicans* has been the species most frequently associated with candidiasis. However, in recent years, a significant increase in infections caused by non-albicans *Candida* species has been observed, a trend likely associated with reduced susceptibility to commonly used antifungal agents (Gonzalez-Lara and Ostrosky-Zeichner, 2020).

*Candida* species are major causes of healthcare-associated infections worldwide, with *Candida auris* emerging as a critical priority due to its resistance and outbreak potential (Dong et al., 2026). *Candida auris* (*C. auris*, recently proposed as *Candidozyma auris*) has emerged as a major public health concern since its first description in 2009 (Satoh et al., 2009; Liu et al., 2024). Shortly after its identification, the species was rapidly isolated across multiple continents, causing simultaneous and independent outbreaks in Asia, Africa and the Americas (da Silva et al., 2025; Lockhart, 2019). Its exceptional ability to persist in healthcare environments, disseminate between patients, and resist standard disinfection procedures has contributed to numerous hospital outbreaks worldwide (Lockhart, 2019; Garcia-Bustos et al., 2026; CDC, 2019; World Health Organization [WHO], 2022). Compounding these challenges, *C. auris* may exhibit resistance to one or more major antifungal classes, with some isolates displaying multidrug-resistant or even pan-resistant profiles, greatly limiting therapeutic options (da Silva et al., 2025; Bastos et al., 2025). These characteristics have led the World Health Organization to classify *C. auris* as a “critical priority” fungal pathogen, and the U.S. Centers for Disease Control and Prevention (CDC) to designate it as an urgent threat, underscoring the global importance of improving its early detection and containment (CDC, 2019, 2022; World Health Organization [WHO], 2022).

Identification of *C. auris*, as well as other yeast species, can be performed using chromogenic media, molecular techniques (e.g., PCR, DNA sequencing, and the T2 *Candida* Panel), and automated systems such as MALDI-TOF MS (Xue et al., 2024; Hsu and Yassin, 2025; Clancy and Nguyen, 2018). However, many of these methods are limited by their high cost. Conventional identification methods, although less expensive, may lack sufficient accuracy, an issue that is particularly critical for *C. auris*, which is frequently misidentified or remains undetected by standard manual protocols (Bongomin and Denning, 2025).

This difficulty is largely explained by its phylogenetic proximity to species within the *Candida haemulonii* complex (*C. haemulonii*, *C. pseudohaemulonii*, and *C. duobushaemulonii*, also known as *Candidozyma haemuli*, *Candidozyma pseudohaemuli*, and *Candidozyma duobushaemuli*), which share more than 80% rDNA sequence similarity and exhibit overlapping physiological characteristics (Ahmad and Alfouzan, 2021).

Given these limitations, near-infrared spectroscopy (NIRS), combined with multivariate analysis techniques (such as PCA-LDA, SPA-LDA, and GA-LDA), has emerged as a promising alternative for the identification of pathogenic yeast species, including *C. auris*. This approach offers several advantages for microbial identification from cultured isolates, including rapid analysis, high diagnostic accuracy, and the elimination of the need for expensive reagents (Nascimento et al., 2024; Medeiros et al., 2026).

Near-infrared spectroscopy (or NIR) analyzes samples by emitting electromagnetic radiation in the near-infrared range (780–2500 nm), enabling interactions between radiation and molecular bonds, where each molecule presents a characteristic absorption pattern (Medeiros et al., 2026). When applied to microbiology, NIRS enables rapid and precise identification of pathogens isolated from clinical or hospital environments (Nascimento et al., 2024). Although traditionally used for the analysis of inorganic substances such as minerals, the development of multivariate analytical models has expanded its application to organic samples, including the discrimination between *C. auris* and *C. haemulonii* (Essendoubi et al., 2005; De Bruyne et al., 2018). However, to date, NIR-based models capable of simultaneously distinguishing multiple pathogenic yeasts from *C. auris* have not been fully explored.

The application of NIRS for the simultaneous discrimination of multiple clinically relevant yeast species, including *C. auris*, remains underexplored despite advances in spectroscopic methods, due to a lack of robust models capable of distinguishing *C. auris* from a broader panel of pathogenic yeasts and related species. Therefore, it is necessary to fill this gap to improve rapid and accurate fungal diagnosis. Thus, the objective of this study was to develop and validate an approach based on NIRS combined with multivariate analysis to differentiate seven clinically important yeast species, focusing on *C. auris*.

## Materials and methods

### Microorganisms and culture

Seven yeast strains belonging to *Candida* and formerly *Candida* species were used in this study. They represent the main yeast species frequently isolated from clinical cases and include reference strains from internationally recognized culture collections, such as the American Type Culture Collection (ATCC) and the Westerdijk Fungal Biodiversity Institute (CBS), as well as well-characterized clinical isolates from previous studies (Ramos et al., 2015; Bastos et al., 2025).

The reference strains included *C. auris* CBS 10913, *C. albicans* ATCC 90029, *N. glabrata* ATCC 2001, *C. parapsilosis* ATCC 22019. In addition, clinical isolates were included: *C. tropicalis* 51L and *Pichia kudriavzevii* AMB18L1, both obtained from Bastos et al. (2025), as well as *Candida haemuloni* CH2, as described by Ramos et al. (2015).

The cultures were maintained in BHI (Brain Heart Infusion) medium supplemented with 10% of glycerol at −80 °C until their use.

#### Cultivation of the yeasts

Glicerol stored strains of the seven species tested were retrieved from storage and thawed at room temperature. An aliquot of 10 μL from each thawed suspension was inoculated into 1 mL of Sabouraud Dextrose Medium (SD) (Kasvi, Brazil) and incubated at 37 °C for 24 h. Following, the culture was streaked on Sabouraud Dextrose Agar (SDA) for additional growth for 24 h at 37 °C.

For the experimental assays, a single colony from each culture was streaked on SDA. Inoculation was performed using the multiple streak exhaustion technique to obtain well-isolated colonies. Each strain was plated on 15 independent agar plates. All plates were incubated at 37 °C for 72 h. The applied methodology was adapted from Nascimento et al. (2024).

#### Acquisition of near-infrared spectra

To ensure the independence of the biological replicates and avoid pseudo-replication, a triplicate was performed by selecting three distinct, well-isolated colonies from 15 independent agar plates per species to capture both intra and inter plate spectral variability. Each selected colony was treated as an independent biological sample for spectral acquisition, resulting in a total of 315 spectra (45 per species) Measurements were performed using a Fourier Transform spectrometer, ARCspectro ANIR (Arcoptix, Switzerland) (Nascimento et al., 2024). Spectral acquisition was performed in reflectance mode with a transflection probe using a 99% reflectance reference placed beneath the samples, covering the spectral range from 900 to 2.600 nm. Prior to sample analysis, a blank measurement was performed using a Petri dish containing only the sterile culture medium. This background signal was subtracted from all yeast sample spectra to eliminate interference from the agar and the baseline laboratory environment. The detector gain was set to the extreme level and spectra were collected using a single scan. A Boxcar smoothing filter was applied at 10-nm intervals, and measurements were performed in triplicate on isolated colonies to capture variability both within the same sample and among different samples.

#### NIR spectra pre-processing

Smoothing using the Savitzky–Golay (SG) algorithm was applied to reduce random noise while preserving relevant spectral information. In addition, multiplicative scatter correction (MSC) was used to correct light scattering effects while maintaining the original spectral shape and scale (Morais et al., 2020). The final portion of the spectra was cut out before performing multivariate analysis, as such part had very poor signal to noise ratio, which could contribute to non-relevant or harmful information for the models. Resulting in spectra from 900 to 2404 nm.

### Multivariate analysis

#### Multivariate analysis of infrared data

Data import, preprocessing, exploratory analysis by PCA and construction of multivariate classification models (SPA-LDA and GA-LDA) were performed using MATLAB software (MathWorks Inc., Natick, MA, USA). Seventy percent of the samples were allocated for model training, fifteen percent for validation and the last fifteen percent for testing, applying the Kennard–Stone algorithm (Nascimento et al., 2024; Kennard and Stone, 1969) to the infrared spectra. Training and validation samples were used for model construction and optimization, while test samples were employed to evaluate classification performance. Sensitivity and specificity analyses were applied to assess model accuracy.

Principal Component Analysis (PCA) is a powerful chemometric method in which the original variables are reduced and combined to form principal components (Morais et al., 2020). These components are linear combinations of the original variables, whereby the spectral matrix X is decomposed:


$$X=T\,P^{t}+E$$

where X is the *I × J* data matrix, T is the *I × A* score matrix, P is the *J × A* loading matrix (variable weights), and E is the *I × J* residual matrix. Here, *I* represents the number of objects, *J* the number of variables, and *A* the number of calculated components. This variable reduction is essential, as a large data set may pose challenges during data classification (Nascimento et al., 2024; Morais et al., 2020).

#### Variable selection algorithms

Variable selection and reduction were performed using two complementary approaches. The Successive Projections Algorithm (SPA) aims to minimize collinearity while selecting variables with minimal redundancy. The Genetic Algorithm (GA) is another approach, employing a probabilistic and non-local search process, analogous to Darwinian “survival of the fittest,” to identify the most relevant variables for model construction (Kennard and Stone, 1969; Sequeira and Borges, 2024).

#### Discriminant analysis

Linear Discriminant Analysis (LDA) is a discriminant analysis algorithm, used for classification, based on calculating the Mahalanobis distance between samples for each class. It is a robust method for handling spectral data (Cheng et al., 2016; Ullah et al., 2021).

A limitation of LDA arises when the number of spectral variables exceeds the number of samples. However, this effect can be mitigated by increasing the sample size according to the central limit theorem, and the application of PCA can overcome this issue, as in the PCA-LDA algorithm, where LDA is applied to the PCA scores (Soares et al., 2013; Qu and Pei, 2024).

In the SPA-LDA and GA-LDA models, overfitting may occur. To address this, a validation set can be used to guide variable selection, allowing the determination of an optimal number of variables for SPA-LDA and GA-LDA. This is achieved through the cost function G, which is calculated for a given validation dataset, as shown in the equation below (Nascimento et al., 2024):


$$G=\frac{1}{N_{v}}\,\overset{N_{v}}{\underset{n=1}{\sum }}g_{n}$$

where Nv is the number of validation samples, and g_n_ is defined as shown in the equation:


$$g_{n}=\frac{r^{2}\,\left(x_{n},m_{I\,\left(n\right)}\right)}{\min_{I\,\left(m\right)\neq I\,\left(n\right)}\,r^{2}\,\left(x_{n},m_{I\,\left(n\right)}\right)}$$

where *I(n)* is the true class index for the n-th validation object *x*_n_. The numerator, *r^2^(x_n_)*, represents the squared Mahalanobis distance between the object *x*_n_ and the mean of its true class, while the denominator corresponds to the squared Mahalanobis distance between *x*_n_ and the error center of the nearest incorrect class (i.e., the misclassified class) (Nascimento et al., 2024).

The classification of the LDA score (L_ij_) is determined as follows:


$$L_{i\,k}={\left(x_{i}-{\bar{x}}_{k}\right)}^{T}\,\sum_{p\,o\,o\,l\,e\,d}^{-1}\left(x_{i}-{\bar{x}}_{k}\right)-2\,l\,o\,g_{e}\,\pi_{k}$$

where x_i_ is an unknown measurement vector for sample *i*, k′ is the measurement vector corresponding to the mean of class *k*, Σ_pooled_ is the pooled covariance matrix, and π_k_ is the prior probability of class *k* (Nascimento et al., 2024).

#### Model performance evaluation

Model performance was evaluated using sensitivity (SENS), specificity (SPEC), and accuracy as key quality parameters (Morais et al., 2020). Sensitivity is defined as the probability of obtaining a positive result for a sample belonging to the class. Specificity is defined as the probability of obtaining a negative result for a sample not belonging to the class. Accuracy represents the proportion of correctly classified samples, considering both true positives and true negatives. These metrics were calculated to assess the classification performance of all models.


$$Accuracy\left(\%\right)=\frac{\mathrm{TP}+\mathrm{TN}}{\mathrm{TP}+\mathrm{FP}+\mathrm{TN}+\mathrm{FN}}\times 100$$

$$SENS\left(\%\right)=\left(\frac{\mathrm{TP}}{\mathrm{TP}+\mathrm{FN}}\right)\times 100$$

$$SPEC\left(\%\right)=\left(\frac{\mathrm{TN}}{\mathrm{TN}+\mathrm{FP}}\right)\times 100$$

Where FN is defined as false negative and FP as false positive. TP refers to true positive and TN to true negative (Morais and Lima, 2018; Morais et al., 2020).

In all calculations, *C. auris* samples were considered the positive class, while all other classes were treated as the negative class.

## Results

**FIGURE 1:**
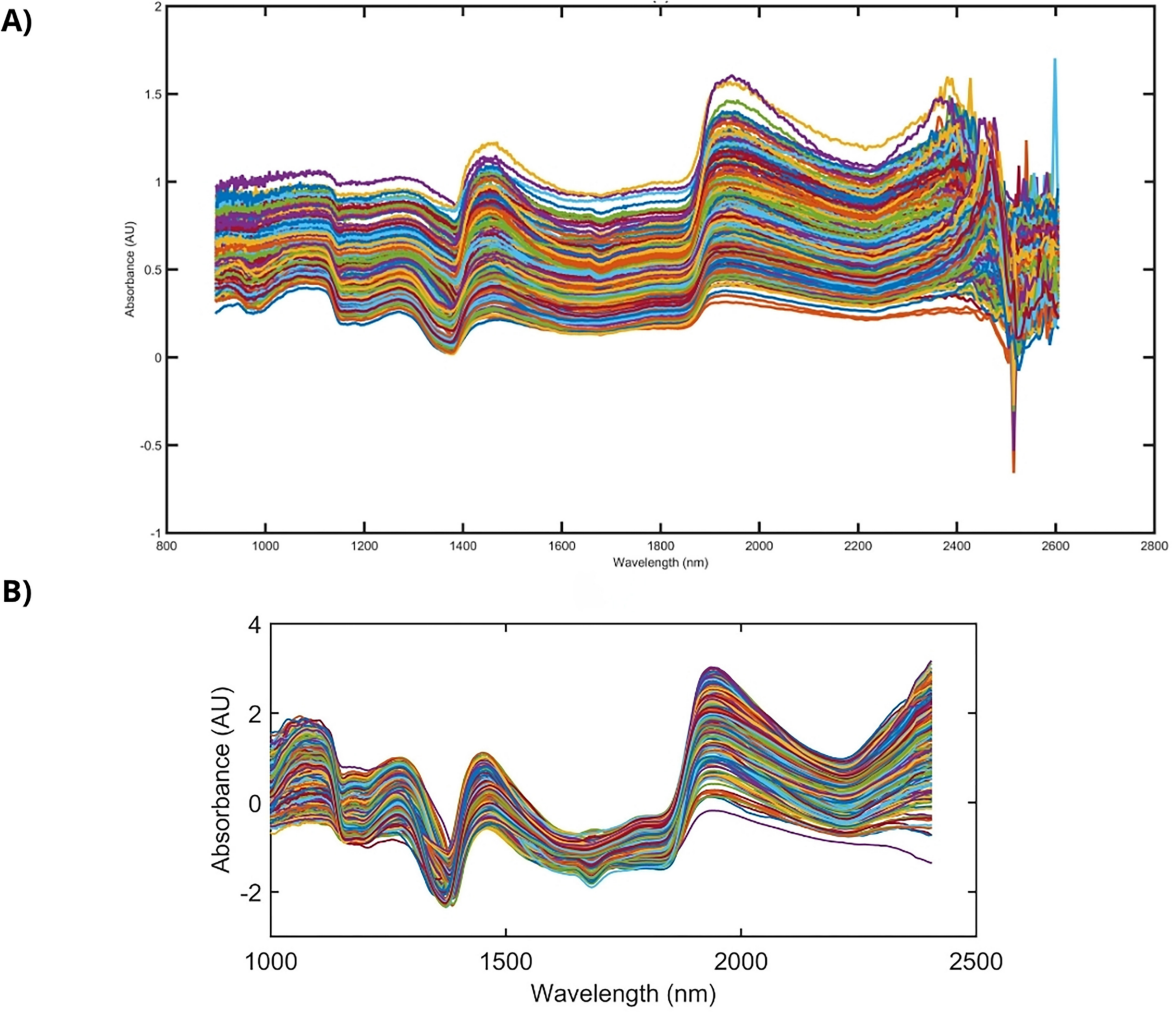
**(A)** Original near-infrared (NIR) spectra obtained from samples belonging to seven yeast species. Measurements were carried out in the spectral range of 900–2600 nm using diffuse reflectance spectroscopy. **(B)** Preprocessed spectra of seven yeast classes obtained by NIR spectroscopy. Data were preprocessed for noise correction and normalization, allowing better comparison among the spectra.

Raw spectra obtained from isolated colonies of *C. auris*, *C. albicans*, *N. glabrata*, *C. tropicalis*, *Pichia kudriavzevii*, *C. parapsilosis*, and *C. haemulonii* are presented in Figure 1A. These species were included in all NIRS analyses. Spectral preprocessing was applied to the raw data to remove or reduce signals unrelated to the analytical objective and to minimize chemically irrelevant variations, thereby improving the precision and accuracy of qualitative and quantitative analyses (Figure 1B).

After preprocessing, a high degree of spectral similarity was observed among the seven species (Figure 1B). Then, computational analyses were applied to identify the markers that differentiate the species.

**FIGURE 2:**
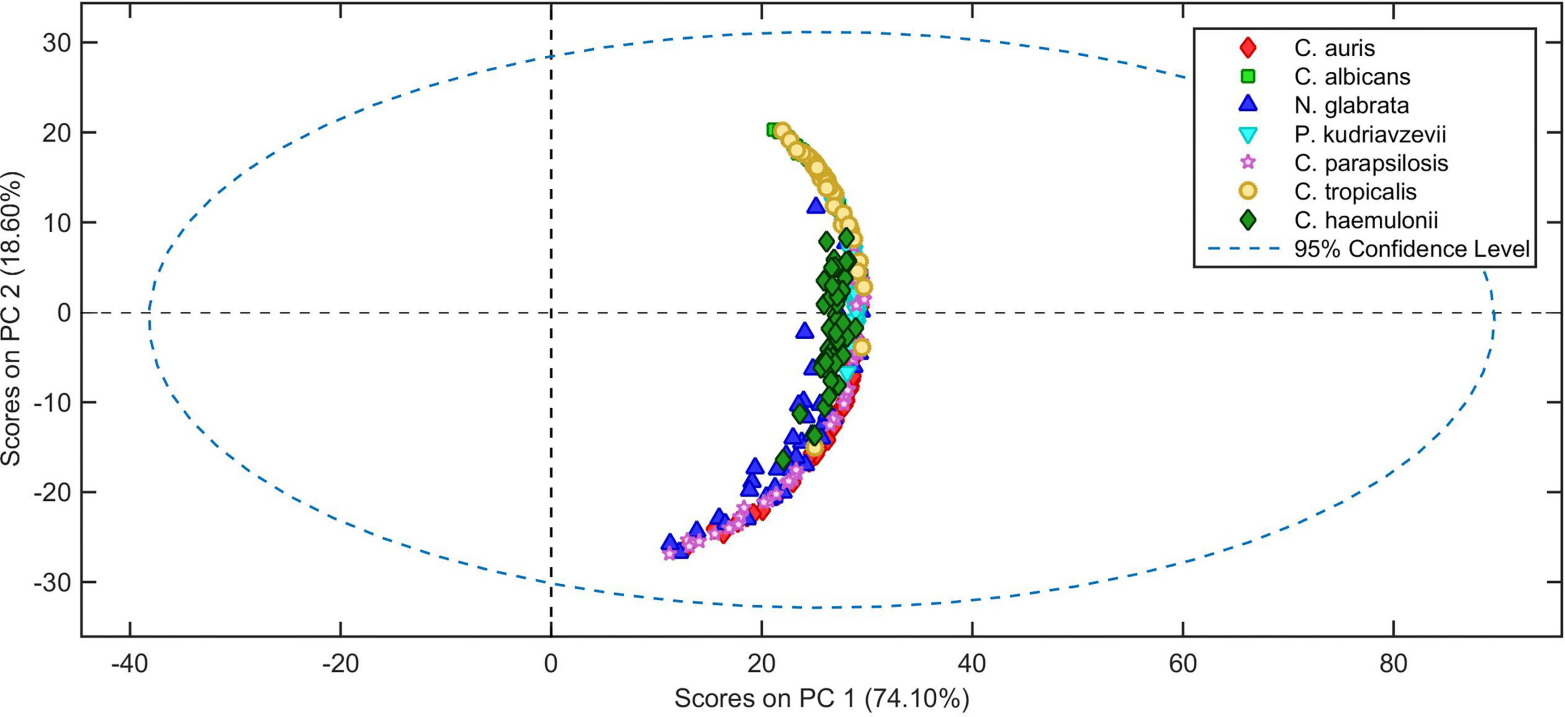
Score plot obtained from PCA of seven *Candida* classes based on NIR spectral data. Each color represents a distinct yeast class, highlighting the clustering among species according to their spectral characteristics.

The NIR data were initially analyzed using PCA to evaluate sample behavior and cluster formation without prior class information. The first two principal components accounted for 92% of the explained variance; however, distinct group formation was not observed (Figure 2). The figure depicts a U-shaped or arch-shaped distortion known as the “horseshoe effect.”

Since PCA generates a transformed space that may obscure original spectral information (Nascimento et al., 2024), the SPA and GA algorithms were applied to select the most relevant variables (i.e., wavelengths). These variables were subsequently used to construct multivariate classification models capable of correctly assigning new samples to their respective classes. For model development, 70% of the data were used for training, 15% for validation, and 15% for testing, applying the KS algorithm (Kennard and Stone, 1969).

The SPA-LDA and GA-LDA combinations successfully classified all yeast species with 100% sensitivity and specificity, effectively separating *C. auris* from other yeast species.

**FIGURE 3:**
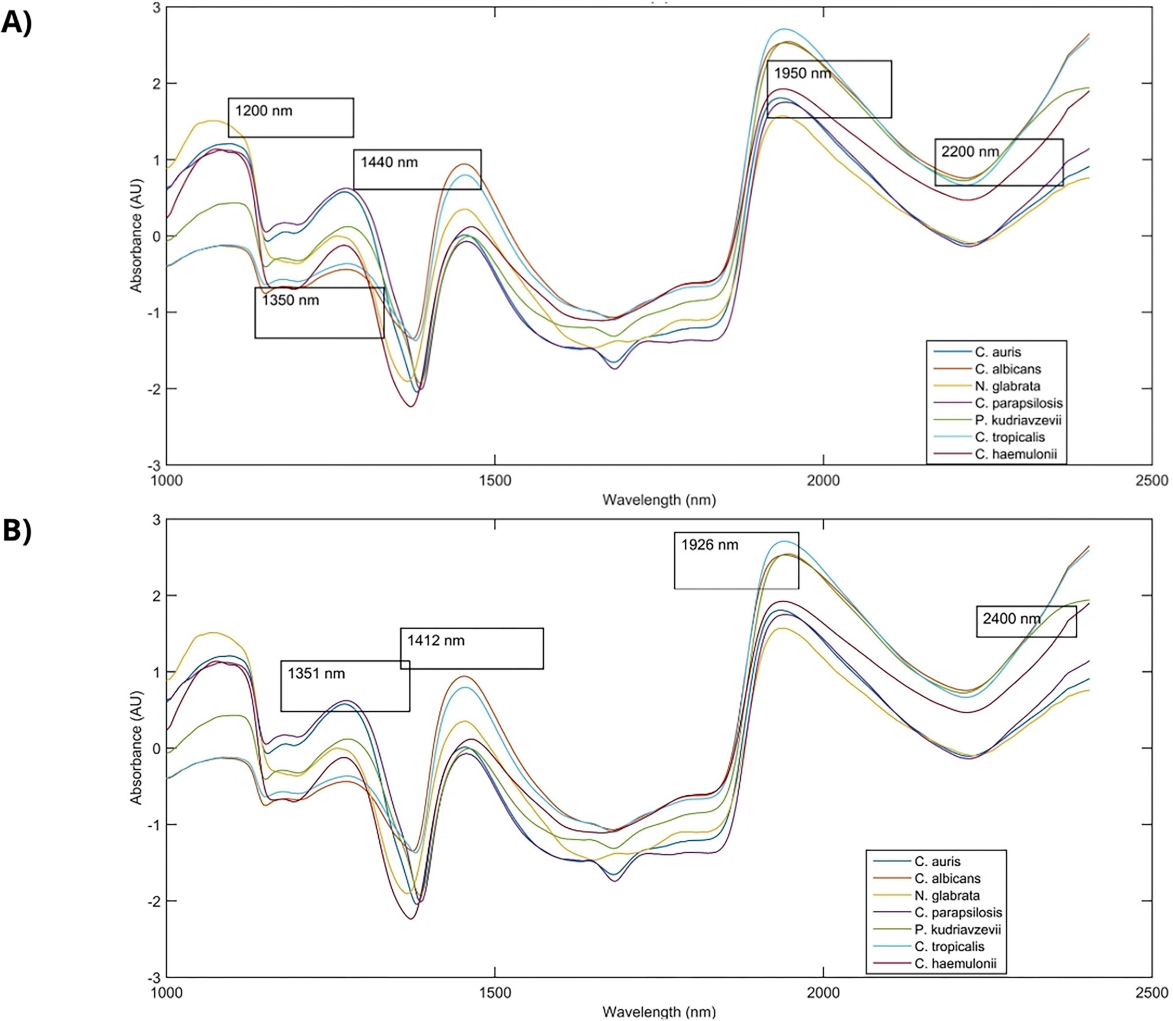
**(A)** Plot of variables selected by the SPA-LDA method in preprocessed spectra of seven yeast classes obtained by NIR spectroscopy. Each spectrum represents the mean of a distinct yeast class. **(B)** Plot of variables selected by the GA-LDA method in preprocessed spectra of seven yeast classes obtained by NIR spectroscopy. Each spectrum represents the mean of a distinct yeast class.

Some wavelengths, such as 1200, 1350, 1440, 1950, and 2200 nm, were selected by the SPA-LDA algorithm (Figure 3A). These spectral regions are associated with C–H and C = O (aldehyde) combination bands, N–H bonds of primary and secondary amides, S–H bonds of thiol groups, and C–H stretching and bending combination bands in aliphatic chains.

In turn, the GA-LDA algorithm selected the wavelengths 1412, 1351, 1926, and 2400 nm (Figure 3B). These regions are mainly related to the first overtone of O–H combination bands of water, as well as overtones and combinations involving C–H vibrations and O–H/C–H interactions. These bands are associated with both water content and functional groups present in cellular biomolecules (Nascimento et al., 2024; Medeiros et al., 2026).

The predictability of the models was evaluated based on the classification of 45 spectra from individual colonies of each isolate. Sensitivity assessed the models’ ability to correctly identify colonies belonging to *C. auris*, while specificity evaluated the models’ ability to correctly identify spectra from other yeast species. Accuracy represented the proportion of samples correctly classified for each species (Nascimento et al., 2024; Medeiros et al., 2026). Maximum performance across all parameters is presented in Table 1.

**TABLE 1:** Performance metrics of SPA-LDA and GA-LDA models for classification of *C. auris* versus other yeast classes.

Results	SPA-LDA	GA-LDA
Sensitivity (%)	100	100
Specificity (%)	100	100
Accuracy (%)	100	100

In addition, a confusion matrix (Table 2) was constructed to identify specific classification errors. It shows how each sample was classified and provides a clear visualization of any misclassifications.

**TABLE 2:** Confusion matrix for SPA-LDA and GA-LDA classification models.

True/ Predicted	*C.* .*auris*	*C. albicans*	*N. glabrata*	*C. parapsilosis*	*P. kudriavzevii*	*C. tropicalis*	*C. haemulonii*
*C. auris*	7	0	0	0	0	0	0
*C.* .*albicans*	0	7	0	0	0	0	0
*N. glabrata*	0	0	7	0	0	0	0
*C. parapsilosis*	0	0	0	6	0	0	0
*P. kudriavzevii*	0	0	0	0	7	0	0
*C. tropicalis*	0	0	0	0	0	7	0
*C. haemulonii*	0	0	0	0	0	0	7

**FIGURE 4:**
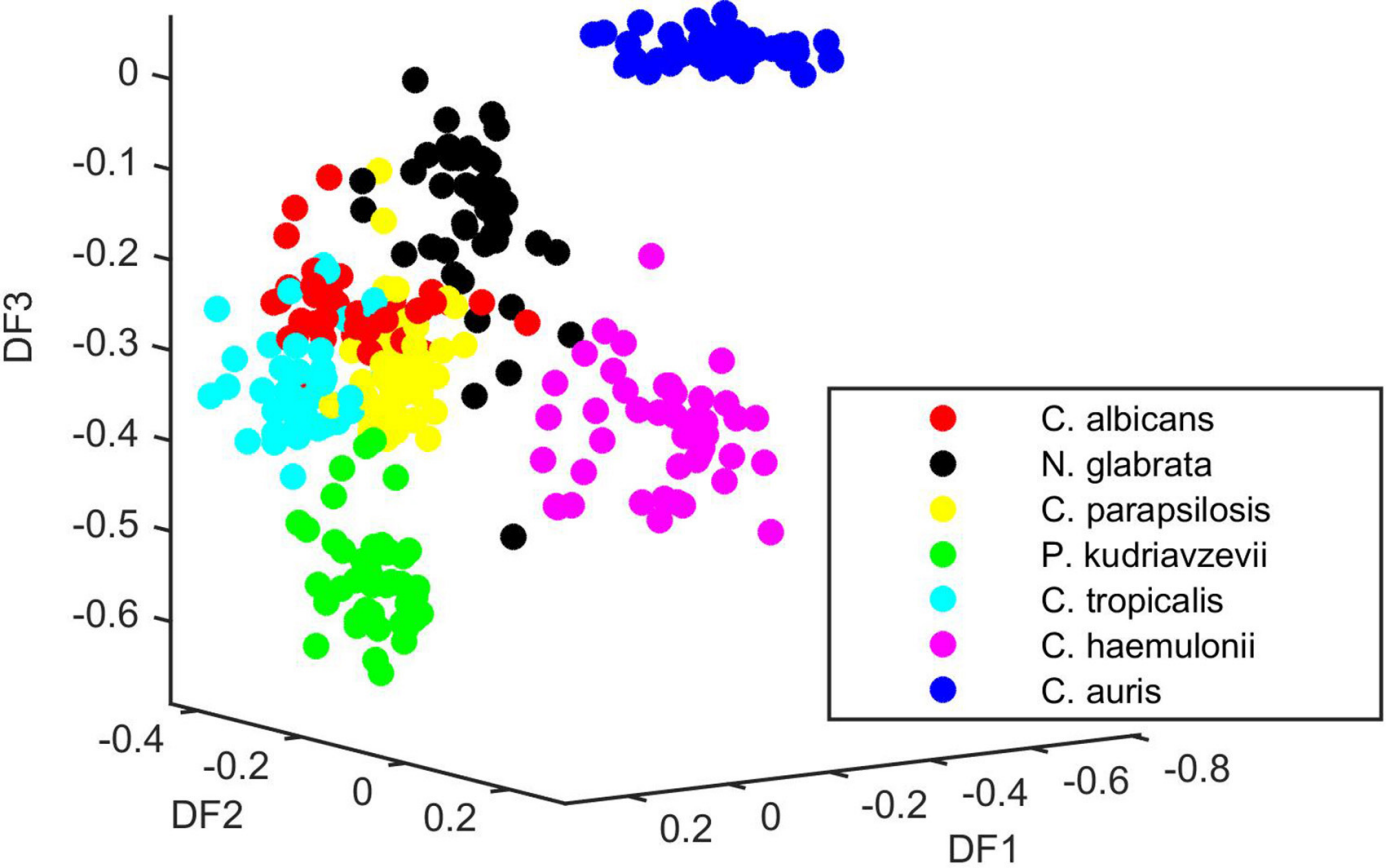
3D plot of the discriminant function obtained for seven yeast classes, showing clustering and separation among species. *C. auris* is clearly distinguished from the other species.

Finally, the differentiation of *C. auris* from other species is shown in a 3D discriminant function plot (Figure 4). The species is clearly grouped and separated from the others, confirming the potential of NIR spectroscopy for rapid and accurate identification e discrimination between the seven of the most pathogenic yeasts species.

## Discussion

Infrared spectroscopy provides detailed information about the cellular composition of microorganisms, functioning as a molecular fingerprint. Each microorganism generates a unique spectral signature due to variations in cell wall structure, membrane composition, and intracellular biomolecules (Muchaamba and Stephan, 2024). Based on this principle, we collected and compared spectral signatures from seven *Candida* and previously *Candida* classified species, enabling their differentiation and classification.

Principal component analysis was initially applied as an unsupervised approach to explore natural clustering patterns without prior class information. However, the data exhibited a horseshoe (arch) effect, and no clear group separation was observed. This phenomenon is commonly associated with non-linear relationships that cannot be adequately captured by linear projection methods. Although often interpreted as an artifact, the horseshoe effect may also reflect intrinsic data structure, indicating that alternative approaches, such as non-linear methods or different data transformations, may be more suitable for this type of dataset (Lewis and Menzies, 2015; Morton et al., 2017).

To overcome this limitation, variable selection methods were applied prior to supervised classification. SPA selected wavelengths around 1200, 1350, 1440, 1950, and 2200 nm, regions associated with combination bands of C–H and C = O groups, N–H vibrations of amides, S–H bonds, and vibrational modes of aliphatic chains. These spectral features are directly linked to key biochemical components of fungal cells, such as polysaccharides, proteins, and other metabolites. Thus, the observed spectral differences among species likely reflect variations in cell wall composition, metabolic profiles, and protein structures (Nascimento et al., 2024; Medeiros et al., 2026; Morais et al., 2020).

Similarly, GA selected wavelengths at approximately 1351, 1412, 1926, and 2400 nm, which are predominantly associated with O–H overtones and combination bands, as well as interactions involving C–H groups. These regions may reflect both intracellular water content and functional groups present in cellular biomolecules (Nascimento et al., 2024; Medeiros et al., 2026).

Notably, some wavelengths selected by both approaches are in similar spectral regions, particularly those related to C–H vibrations, suggesting that these regions contain relevant discriminatory information. At the same time, the differences in selected variables indicate that each method captures distinct yet complementary spectral features. Together, these findings reinforce that multiple regions of the spectrum contribute to species-level discrimination.

Collectively, the selected wavelengths correspond to subtle but significant biochemical differences among the seven species, which can be attributed to variations in cell wall polysaccharides, peptide and protein structures, and metabolic products (Neves et al., 2016; Workman and Weyer, 2008). When coupled with the selected variables, LDA demonstrated a high capacity to discriminate among the seven yeast species. Both SPA-LDA and GA-LDA models achieved 100% sensitivity, specificity.

Although the classification models achieved the highest levels of sensitivity, specificity, and accuracy in this study, we acknowledge that the use of a single reference or clinical strain per species is a significant limitation. A single strain cannot fully reflect the intraspecific genetic and phenotypic heterogeneity inherent in pathogenic yeasts. Therefore, this work should be interpreted as a robust proof-of-concept demonstrating the discriminatory potential of FT-NIR spectroscopy. Future validations incorporating a broader collection of clinical isolates from diverse geographical and clinical sources (e.g., blood, urine) and different media are necessary to confirm the generalizability of these models (Medeiros et al., 2026).

The high performance observed for the SPA-LDA and GA-LDA models can be attributed to the rigorous standardization of experimental conditions (time of incubation, temperature and medium), selection of well characterized reference strains and the use of the KS algorithm in selecting 70% of the samples for calibration, 15% for validation, and 15% for testing serving as an initial, yet robust, model to evaluate the discriminatory capabilities of NIR. By selecting representative samples for the training, validation, and test sets, the KS algorithm minimizes data leakage and helps the model to evaluate better the spectral information. Furthermore, the application of already mentioned variable selection algorithms reduced collinearity and redundancy, focusing only on the most informative wavelengths for species discrimination, which significantly mitigates the risk of overfitting. These results highlight the robustness of the models and indicate that the selected spectral features were highly informative for classification.

The potential of infrared spectroscopy for microbial identification has been widely demonstrated. However, most studies have focused on mid-infrared spectroscopy combined with multivariate analysis to distinguish *Candida* isolates (Potocki et al., 2019; Lam et al., 2022; Franconi et al., 2025). Notably, Vatanshenassan et al. (2020) demonstrated that infrared spectroscopy can reliably identify and cluster emerging yeasts such as *C. auris*, showing performance comparable to established methods, including microsatellite typing, ITS sequencing, AFLP fingerprinting, and MALDI-TOF mass spectrometry. Although each technique presents distinct advantages and limitations, infrared spectroscopy showed strong discriminatory power (Vatanshenassan et al., 2020).

More recently, FT-IR has been applied to differentiate *C. auris* clades and investigate epidemiological relationships during outbreak scenarios. de Melo et al. (2025) demonstrated the ability of FT-IR to discriminate clades I, II, III, and IV and to identify biochemical similarities among isolates from different hospitals, particularly in spectral regions associated with polysaccharides and lipids. These findings highlight the potential of vibrational spectroscopy not only for species identification but also for epidemiological studies (de Melo et al., 2025).

Despite these advances, the application of NIR in fungal identification remains limited. Nascimento et al. (2024) demonstrated the effectiveness of NIR combined with multivariate analysis for distinguishing *C. auris* from *C. haemulonii* sensu stricto, achieving 100% sensitivity and specificity. The landscape of pathogenic yeast identification is currently dominated by MALDI-TOF MS and molecular-based methods, each presenting distinct trade-offs in clinical practice. MALDI-TOF MS is widely regarded as a gold standard due to its high accuracy and standardized workflows; however, its implementation is often hampered by high initial capital investment and substantial maintenance costs, alongside a reliance on proprietary proteomic databases (Zhao et al., 2024). Similarly, molecular techniques like PCR and DNA sequencing provide unrivaled sensitivity but are limited by the need for specialized laboratory infrastructure, expensive reagents, and time-consuming sample preparation (Morovati et al., 2023). In this context, NIRS coupled with multivariate analysis offers a transformative alternative. It enables rapid, reagent-free identification with minimal sample processing, significantly reducing operational costs per sample (Medeiros et al., 2026). Nevertheless, while NIRS excels in speed and cost-efficiency, its diagnostic reliability remains critically dependent on the robustness of chemometric models and the breadth of the training datasets, whereas MALDI-TOF and molecular assays benefit from more globally established standardization.

In this context, the present study expands the application of NIRS by demonstrating its ability to accurately discriminate seven clinically relevant yeast species. Furthermore, the greater penetration depth of NIR radiation, combined with chemometric analysis, enhances its applicability for microbial classification. Notably, the method successfully discriminated *C. auris* from closely related species such as *C. haemulonii*, which is of particular clinical relevance. The accurate identification of *C. auris* remains a major challenge in clinical microbiology due to frequent misidentification by conventional methods, yet it is essential given its multidrug resistance, high transmissibility, and association with healthcare-associated outbreaks. Therefore, the ability of this approach to reliably distinguish *C. auris* represents a significant advance with potential implications for infection control and patient management.

In conclusion, we showed that FT-NIR spectroscopy combined with multivariate analysis, particularly SPA-LDA and GA-LDA, demonstrated a strong potential for the accurate identification of seven yeast species: *C. auris*, *C. albicans*, *N. glabrata*, *Pichia kudriavzevii*, *C. parapsilosis*, *C. tropicalis*, and *C. haemulonii*, achieving 100% accuracy, sensitivity, and specificity.

## Data Availability

The raw data supporting the conclusions of this article will be made available by the authors, without undue reservation.
